# Generation and Characterisation of Friedreich Ataxia YG8R Mouse Fibroblast and Neural Stem Cell Models

**DOI:** 10.1371/journal.pone.0089488

**Published:** 2014-02-21

**Authors:** Chiranjeevi Sandi, Madhavi Sandi, Harvinder Jassal, Vahid Ezzatizadeh, Sara Anjomani-Virmouni, Sahar Al-Mahdawi, Mark A. Pook

**Affiliations:** Ataxia Research Group, Division of Biosciences, School of Health Sciences and Social Care, Brunel University, Uxbridge, United Kingdom; National Institute for Medical Research, Medical Research Council, London, United Kingdom

## Abstract

**Background:**

Friedreich ataxia (FRDA) is an autosomal recessive neurodegenerative disease caused by GAA repeat expansion in the first intron of the *FXN* gene, which encodes frataxin, an essential mitochondrial protein. To further characterise the molecular abnormalities associated with FRDA pathogenesis and to hasten drug screening, the development and use of animal and cellular models is considered essential. Studies of lower organisms have already contributed to understanding FRDA disease pathology, but mammalian cells are more related to FRDA patient cells in physiological terms.

**Methodology/Principal Findings:**

We have generated fibroblast cells and neural stem cells (NSCs) from control Y47R mice (9 GAA repeats) and GAA repeat expansion YG8R mice (190+120 GAA repeats). We then differentiated the NSCs in to neurons, oligodendrocytes and astrocytes as confirmed by immunocytochemical analysis of cell specific markers. The three YG8R mouse cell types (fibroblasts, NSCs and differentiated NSCs) exhibit GAA repeat stability, together with reduced expression of frataxin and reduced aconitase activity compared to control Y47R cells. Furthermore, YG8R cells also show increased sensitivity to oxidative stress and downregulation of *Pgc-1α* and antioxidant gene expression levels, especially *Sod2*. We also analysed various DNA mismatch repair (MMR) gene expression levels and found that YG8R cells displayed significant reduction in expression of several MMR genes, which may contribute to the GAA repeat stability.

**Conclusions/Significance:**

We describe the first fibroblast and NSC models from YG8R FRDA mice and we confirm that the NSCs can be differentiated into neurons and glia. These novel FRDA mouse cell models, which exhibit a FRDA-like cellular and molecular phenotype, will be valuable resources to further study FRDA molecular pathogenesis. They will also provide very useful tools for preclinical testing of frataxin-increasing compounds for FRDA drug therapy, for gene therapy, and as a source of cells for cell therapy testing in FRDA mice.

## Introduction

FRDA is an autosomal recessive neurodegenerative mitochondrial disorder caused primarily by a homozygous GAA repeat expansion mutation within intron 1 of the frataxin (*FXN*) gene, located on chromosome 9q21.1 [1]. Approximately 4% of FRDA patients are compound heterozygotes, having a GAA repeat expansion on one allele and an inactivating or loss-of-function *FXN* gene mutation, such as a point mutation [2], [3] or a deletion/duplication [4]–[6] on the other allele. The prevalence of FRDA is 1–2 in 50,000 in Caucasian populations with an equal occurrence in both genders [7] and an estimated carrier frequency of 1∶60 to 1∶100 [8]. Unaffected individuals have up to 43 GAA repeats, while affected individuals have 44 to 1700 GAA repeats, most commonly between 600–900 GAA repeats [8], [9]. The length of the smaller GAA repeat correlates with FRDA disease severity and inversely correlates with the age of onset [10], [11]. Although the cause of the GAA repeat expansions in FRDA is not fully understood, there is evidence for involvement of abnormal DNA replication, transcription or repair [12]–[14]. The effect of the GAA repeat expansion is to decrease expression of the essential and ubiquitously expressed mitochondrial protein frataxin, with levels in FRDA patients ranging from 4% to 29% that of normal [15]. However, asymptomatic carriers produce about 50% frataxin levels compared to unaffected individuals [16]. Therefore, drugs that induce frataxin expression, at least to the levels of healthy carriers, would be beneficial.

Reduced levels of frataxin in FRDA patients are associated with defects of iron-sulphur (Fe-S) cluster biosynthesis [17], mitochondrial iron accumulation in heart, spinal cord and dentate nucleus [18]–[20], and increased susceptibility to oxidative stress [21]. Pathologically the most obvious effects are loss of large sensory neurons in the dorsal root ganglia (DRG) and degenerative atrophy of the posterior columns of the spinal cord, contributing to symptoms of progressive ataxia, muscle weakness, and sensory deficit. In addition to progressive neurological disability, there is also pathological involvement of non-neuronal tissues, with hypertrophic cardiomyopathy a common feature, and diabetes mellitus identified in approximately 10% of FRDA patients [22]. Skeletal abnormalities such as kyphoscoliosis and pes cavus are also common. At present there is no effective therapy for FRDA, and affected individuals generally die in early adulthood from the associated heart disease. Therefore, there is a high unmet clinical need to develop a therapy for this devastating disorder.

Model systems of human cells and/or non-human cells and organisms can provide insights into FRDA disease pathology. The high evolutionary conservation of frataxin across the species has enabled the development of disease models in various organisms, from the unicellular eukaryote *Saccharomyces cerevisiae* to the complex multicellular mouse model. Depending on the frataxin expression levels, various models of FRDA have shown that different, and even opposite, phenotypes can be observed (reviewed in [23], [24]). Therefore, a combination of studies is needed for the better understanding of the pathophysiological functions of frataxin. With this in mind, several groups have previously developed useful FRDA cell models. For example, to generate a cellular model of a neural lineage, Tan and colleagues transfected human neuronal precursor NT2 (N-tera2) cells with frataxin-specific interfering RNA (RNAi). The resultant cell line showed approximately 70% reduction in *FXN* mRNA and corresponding reduced levels of frataxin protein were found compared with a scrambled RNAi treated cell line [25]. Sarsero and colleagues generated another human cell model with a BAC genomic reporter construct consisting of an in-frame fusion between the human *FXN* gene and EGFP under the control of *FXN* promoter [26]. However, due to the absence of expanded GAA repeats (the construct has 6 GAA repeats) this model only allows the identification of molecules which act on the WT promoter but not on GAA repeats. Grant and colleagues generated an additional GFP reporter cell line by combining part of the first intron of *FXN*, containing either 15 or 148 GAA repeats, to the coding sequence of EGFP [27]. Subsequent analysis of GFP expression levels by fluorescence assay and western blotting demonstrated reduced levels of GFP expression in the [GAA]_148_ cell line compared with the [GAA]_15_ cell line [27], suggesting that these cell lines are appropriate models to study the GAA-mediated silencing effect of *FXN* gene. Similarly, Lufino and colleagues have generated a clonal human cell line by inserting ∼310 GAA •TTC repeats at intron 1 of the *FXN* gene and demonstrated that the insertion of such repeats can recapitulate the epigenetic modifications and *FXN* gene repression, as seen in FRDA patients [28]. Calmels and colleagues have reported the establishment of cellular models based on frataxin missense mutations [29]. In addition, recent reports have described the establishment of human induced pluripotent stem (iPS) cells from FRDA patient fibroblasts [30]–[32], and found *MSH2*-dependent expansion of GAA repeats [30].

None of the currently available cell models reproduce all of the essential molecular and cellular disease mechanisms that are known to occur in FRDA patients. Therefore, there is still a need for the development of further cell models. A good FRDA cell model should have significant reduction of frataxin expression, ideally as a result of transcriptional silencing mediated by a GAA expansion within the genomic context of the frataxin locus. Therefore, we have developed novel fibroblasts, NSCs and differentiated NSCs from a mouse model that has normal-sized GAA repeats (9 repeats, Y47R) [33] and another mouse model with expanded GAA repeats (190+120 repeats, YG8R) [34]. In line with the FRDA-like phenotype, the YG8R mouse cellular models exhibit GAA repeat-mediated *FXN* gene silencing associated with increased DNA methylation, together with reduced levels of aconitase activity and *Pgc-1α* and *Sod2* expression levels. Furthermore, the YG8R cells also show downregulation of several MMR genes, suggesting a potential deficit in the MMR gene machinery that could contribute to an observed GAA repeat stability.

## Materials and Methods

### Animal Procedures and Cell Culture

Primary cells were obtained from schedule 1 culling of two Y47R (9 GAA repeats) and two YG8R (190 GAA repeats) adult mice. The mice were obtained by breeding procedures in accordance with a commitment to replacement, refinement and reduction and no in vivo experiments were performed on these mice. Mice were housed in conventional open cages with Litaspen Premium 8/20 bedding, paper wool nesting and standard fun tunnel environmental enrichment, with 13 hours light, 11 hours dark, 20–23°C and 45–60% humidity. The mice were given a diet of SDS RM3 Expanded food pellets and standard drinking water. All procedures were carried out in accordance with the UK Home Office ‘Animals (Scientific Procedures) Act 1986’ and with approval from the Brunel University Animals Welfare and Ethical Review Board. Two fibroblast cell lines were established from both Y47R and YG8R mouse kidney tissues [33], [34]. The tissues were aseptically collected, chopped into small pieces, followed by enzymatic digestion with trypsin-EDTA (0.25%). Primary cell cultures were grown in DMEM medium with 10% FBS and 1% penicillin-streptomycin (all from Invitrogen) in 5% CO_2_ at 37°C. Two NSC lines were also established from both Y47R and YG8R mice. The mice were sacrificed and the brains were collected carefully in Pg solution (1×PBS, 30% glucose and 1% pen-strep) followed by collecting the sub-ventricular zone (SVZ) using a dissecting microscope. The tissues were minced into small pieces with scalpel blades and digested with a 10 ml papain solution, followed by incubation at 37°C for 60 min on a rocking platform. At the end of the incubation, cells were collected by centrifugation at 800 g for 10 min and almost all the supernatant overlaying the cell pellet was removed carefully, without using suction. The cell pellet was dissociated several times by triturating up and down with p1000 Gilson tips. Cells were resuspended in 7 ml EBSS followed by centrifugation at 800 g for 10 min at room temperature. After centrifugation, the supernatant was discarded and the cell pellet was dissociated with p200 Gilson tips by pipetting up and down 20–30 times. Cells were resuspended in 8 ml EBSS and centrifuged at 600 g for 15 min. The supernatant was discarded and the cell pellet was gently dissociated with a p200 Gilson tip. Finally, the cell pellet was resuspended in 1 ml of complete NSC medium and transferred to a flask containing 7 ml of NSC medium. NSC medium consists of 10% NSC proliferation supplements, 20 ng/ml rhEGF, 10 ng/ml rhFGF (basic) and 2 µg/ml heparin (all from Stem Cell Technologies). Cells were incubated in 5% CO_2_, 95% humidity at 37°C. The differentiation of NSCs was carried out by mechanical dissociation of NSCs with a p200 Gilson tip followed by collection of cells by centrifugation. The dissociated cells were then incubated with NSC differentiation medium on poly-d-lysine coated culture flasks. NSC differentiation medium consisted of NSC basal medium and 10% NSC differentiation supplements (Stem Cell Technologies). The medium was replaced after every two days and differentiated cells were maintained for 7–10 days. The cells were grown in 5% CO_2_ at 37°C.

### Immunocytochemistry

Cells were fixed in 4% paraformaldehyde and permeabilized and incubated with 0.1% Triton X-100 for 15 min. Cells were then incubated with primary antibodies: beta III-tubulin (Abcam) as a marker for neurons, Gal-C (Millipore) as a marker for oligodendrocytes, GFAP (Stem Cell Technologies) as a marker for astrocytes and CD11b (Abcam) as a marker for microglia. All antibodies were incubated at 4°C overnight or 37°C for 2 hours. Secondary antibodies were then added at room temperature incubated for one hour followed by nuclear staining with DAPI.

### GAA Repeat Analysis

Genomic DNA was isolated from 1×10^6^ cells by phenol/chloroform extraction. GAA PCR amplification was carried out with a conventional PCR kit (Qiagen) using 200–500 ng DNA, GAA-F and GAA-R primers and PCR conditions as previously described [1]. GAA PCR products were resolved in 20-cm long 1.5% agarose 1 X TBE gels by electrophoresis at 50 V for 16–20 h and band sizes were determined by comparison with 1 Kb+ and 100 bp DNA size markers (Invitrogen). The number of GAA repeats was then determined by subtracting 451 bp (flanking non-repeat DNA) from the PCR product size, followed by division of the remaining base pair repeat size by 3.

### Quantitative RT-PCR

Total RNA was isolated from approximately 1×10^6^ cells with Trizol (Invitrogen) and cDNA was then prepared by using AMV reverse transcriptase (Invitrogen) with oligo-dT primers. For the *FAST1*expression analysis, the cDNA was synthesised using strand specific primer: FAST-RT: 5′- CCAAGCAGCCTCAATTTGTG-3′ as previously described [35]. The mRNA levels were determined by quantitative RT- PCR analysis using ABI 7900 Fast Real-Time PCR System (Applied Biosystems). TaqMan® gene specific primer pairs and probes were used for *FXN* (Hs00175940_m1), *Catalase* (Mm01340247_m1), *Sod1* (Mm01344232_g1), *Sod2* (Mm00449725_g1), *Gpx1* (Mm00656767_g1), *Gapdh* (Mm99999915_g1) and *β2M* (Mm00437762_m1). The levels of *FAST1*, MMR genes, *Pgc-1α* and *Hprt* were quantified using SYBR® green (Applied Biosystems) with the following set of primers: *FAST1*: N-FAST-F2 5′-GACCCAAGGGAGACTGCAG-3′and 5′- CACTTCCCAGCAAGACAGC-3′, *Msh2*-F 5′-GCAACAACAAGAACTTCAGCACA-3′ and R 5′-CCAAGATGACTGGTCGTACAT-3′, *Msh3*-F 5′-GCCTCAGGGTGGTGAGCTGC-3′ and R 5′-GATCCAGCGCGTCCTCCACG-3′, *Msh6*-F 5′-TTCTCCCTGGCCAAGGTCGCT-3′ and R 5′-TCCCACCCATGTTTGGTCCGGT-3′, *Pms2*-F 5′- ATGGAGCAAACCGAAGGCGTG-3′ and R 5′-GAGGTCGGCAAACTCTTGAA-3′, *Pgc-1α*-F 5′-TGGGGGCACCTGAACAGAACG-3′ and R 5′-GACGGTACCGGAGGCTGACAAC-3′ and *Hprt*-F 5′-AGTCCCAGCGTCGTGATTAG-3′, *Hprt*-R 5′- TTTCCAAATCCTCGGCATAATGA-3′. The mRNA expression levels of *Gapdh* and *β2M* or *Hprt* were also measured in all samples to normalise the gene expression levels to avoid sample-to-sample differences in RNA input, RNA quality and reverse transcription efficiency. Either 1X TaqMan® Universal PCR Master Mix (Applied Biosystems) or 1X Fast SYBR® Green Master Mix (Applied Biosystems) was used along with 1 µl of sample cDNA in 20 µl reaction mixture. PCR conditions were set as10 min at 95°C for enzyme activation followed by 40 two-step cycles (15 sec at 95°C and 1 min at 60°C). Reactions were carried out in triplicate for each biological sample and each experiment was repeated at least two times. Values were expressed relative to *Gapdh* and *β2M* or *Hprt*, and expression levels were calculated by 2^−ΔΔCt^ method and RQ manager software (Applied Biosystems).

### Cell Lysates and Frataxin Protein Dipstick Assay

Approximately 1–3×10^6^ cells were lysed using Cell Lytic™ buffer (Sigma) and protease inhibitor cocktail (40 µl/ml, 25X complete) on ice for 15 min. The extracts were clarified by centrifugation at 10,000 g for 15 min at 4°C and the supernatants were collected. The protein concentration was determined by using the BCA Protein Assay Kit (Pierce). Frataxin protein levels in Y47R and YG8R derived cells were measured by lateral flow immunoassay using Frataxin Dipsticks (Mitosciences), as previously described [36]. Frataxin signal intensities were measured with a Hamamatsu ICA-1000 immuno-chromato reader (Mitosciences).

### MethylScreen Assay

DNA methylation analysis was performed using the ‘MethylScreen’ method [37], which uses combined restriction digestion of DNA with methylation sensitive and methylation dependent restriction enzymes, MSRE and MDRE respectively. MethylScreen was used to analyse the two CpG sites, CpG3 and CpG6 [38], at *FXN* locus upstream of the GAA repeat. 1 µg of genomic DNA was digested with: (1) a MSRE, (2) MDRE, (3) both MSRE and MDRE (double digest, DD), and (4) neither MSRE or MDRE (mock control). The MSREs used for CpGs 3 and 6 were AciI (Fermentas), and Hpy188III (New England Biolabs), respectively [38]. The MDRE used for all two CpGs was McrBc (Fermentas). A 50ng aliquot of digested DNA was then amplified by quantitative PCR using SYBR® Green (Applied Biosystems) and an ABI 7900 Fast Real-Time PCR System (Applied Biosystems) with the following primers: CpG3 F 5′-GAGACGTGGCTTTGTTTTCTG-3′ and R 5′-GTTTCCTCCTTTCAAGCCGTG-3′; CpG6 F 5′-GAAGATGCCAAGGAAGTGGTAG-3′ and R 5′-GAGCAACACAAATATGGCTTGG-3′. PCR quantification was carried out using the ΔCt method (values were calculated as 2^ΔCt^ (mock – digest) with the mock value set at 100%) and RQ Manager software (Applied Biosystems). Each qRT-PCR reaction was performed in triplicate. Methylscreen DNA methylation values were then calculated as follows: Densely methylated (DM) = (MSRE-DD)/(100-DD)×100; unmethylated (UM) = (MDRE-DD)/(100-DD)×100; intermediately methylated (IM) = 100–(DM+UM).

### Aconitase Assay

Aconitase activities were determined using the Aconitase Assay Kit (Cayman Chemical Company, 705502). To perform the assays, cell protein lysates (50 µl) were added to 200 µl of substrate mix (50 mM Tris/HCl pH 7.4, 0.4 mM NADP, 5 mM Na citrate, 0.6 mM MgCl_2_, 0.1% (v/v) Triton X-100 and 1 U isocitrate dehydrogenase) and the reactions were incubated at 37°C for 15 min, followed by spectrophotometric absorbance measurements every minute for 15 min at 340 nm 37°C to determine the reaction slope. Aconitase activities of mouse cells were then normalized to citrate synthase activities, which were determined using a citrate synthase assay kit (Sigma, CS0720).

### Oxidative Stress and PrestoBlue® Cell Viability Assay

Cells were cultured in a 48 well culture plate for 2 days (50% confluence) and washed once with PBS followed by adding fresh medium containing 100 µM of H_2_O_2_ or 100 µg/ml ferric ammonium citrate (FAC) and 1 mM L-buthionine-sulfoximine (BSO). Untreated cells were also grown simultaneously as controls. Cells were incubated for 24 hours and PrestoBlue® reagent (Invitrogen) was added to a 1x final concentration followed by incubating the cells for further 24 hours. Upon entering a living cell, PrestoBlue® reagent is reduced from resazurin, a blue compound with no intrinsic fluorescent value, to resorufin which is red in colour and highly fluorescent. Conversion is proportional to the number of metabolically active cells and therefore can be measured quantitatively. The colour intensity was then measured using xMark™ Microplate Absorbance Spectrophotometer (Bio-Rad) with 570 nm of excitation wavelength and 600 nm emission wavelength. All samples were analysed as 4–6 independent experiments.

### Statistical Analysis

Statistical values comparing two sample groups were determined using the student’s t test and a statistical significance level of *P*≤0.05 was chosen.

## Results

### Generation of Fibroblast and NSC Cultured Cell Lines and Differentiation of NSCs

**Figure 1:**
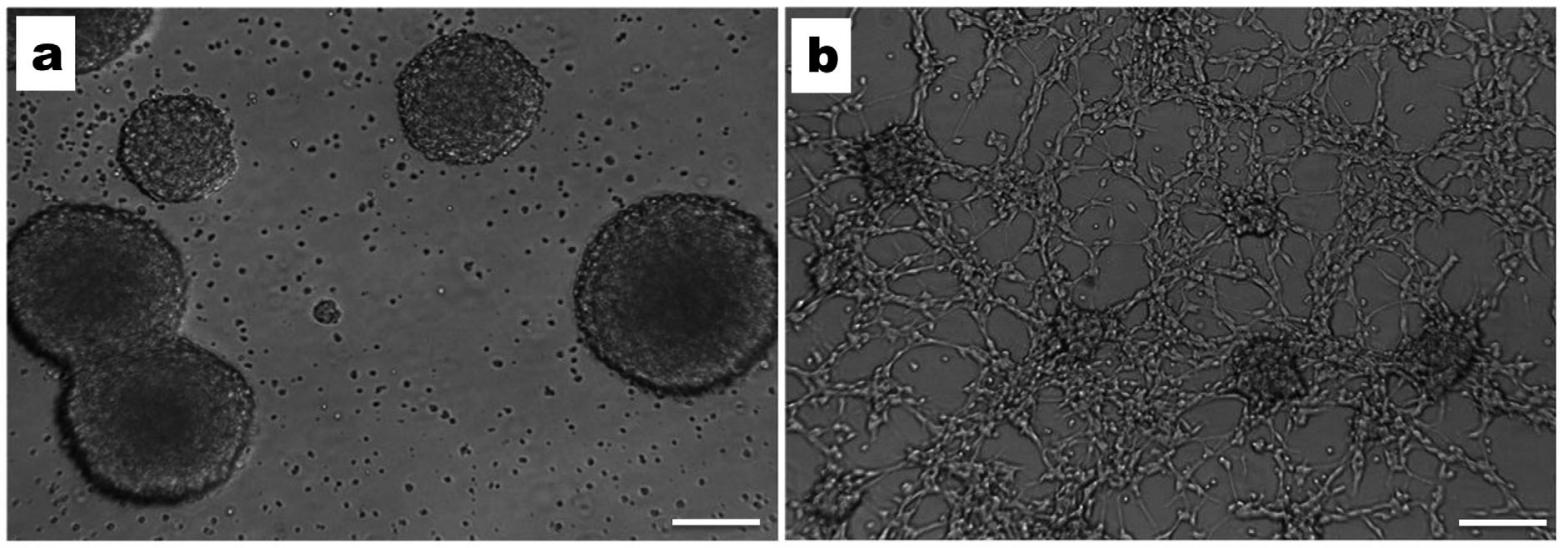
Mouse NSCs and differentiated NSCs in culture. **a**) NSC appeared as “Neurospheres” after 10–14 days of culture with NSC medium supplemented with rhEGF and rhFGF growth factors (10X magnification). **b)** The differentiation of NSCs was induced by incubating the cells in the NSC medium with differentiation supplements. The early stages of the differentiation, where mixed cells are seen emerging from the neurospheres (10X magnification). Scale bars = 100 µm.

**Figure 2:**
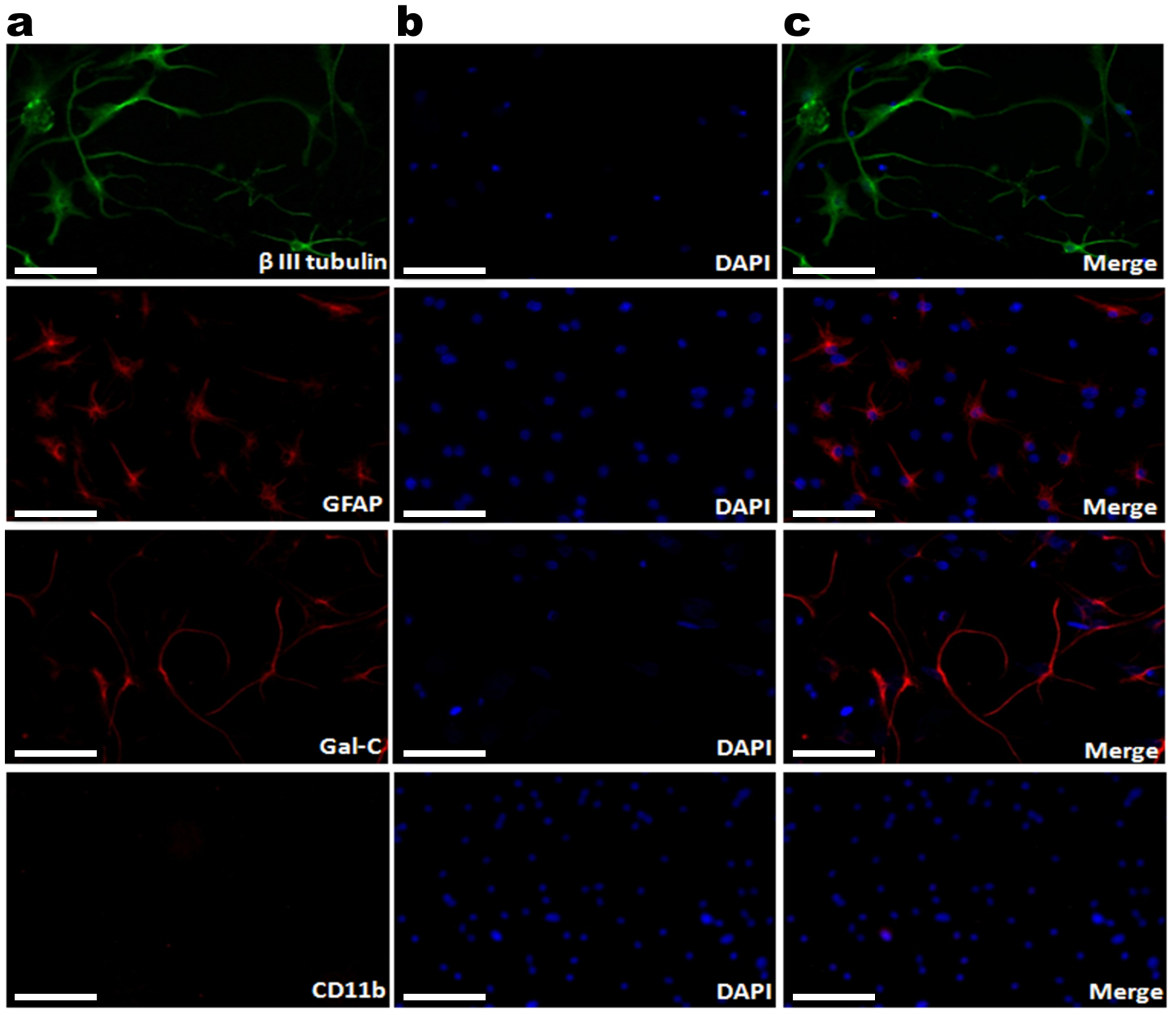
Characterization of differentiated NSCs by immunocytochemistry. (**a**) After 7 days in culture, differentiated NSCs were positively stained with β III-tubulin (neurons), GFAP (astrocytes), and Gal-C (oligodendrocytes) and negatively stained with CD11b (microglia); (**b**) nuclei stained with DAPI, and (**c**) merged images. The images were taken at the magnification of 40X. Scale bars = 25 µm.

To study the effects of the GAA repeat expansion on *FXN* expression in non-CNS and CNS cells, we have established fibroblast and NSC cultured cell lines from adult Y47R control mice (9 GAA repeats) and YG8R FRDA mice (190+90 GAA repeats). Fibroblasts were obtained from freshly isolated kidneys, while NSCs were isolated from the sub-ventricular zone (SVZ) of the brain and dissociated mechanically followed by enzymatic digestion of the tissue. NSCs were maintained in NSC medium with rhEGF and rhFGF growth factors. After 10–14 days in culture, NSCs were observed as free-floating ‘neurospheres’, with each neurosphere containing approximately 200–500 cells (Fig. 1). To determine whether the NSCs were capable of differentiating into neurons, oligodendrocytes and astrocytes, we induced differentiation using either intact NSCs or dissociated NSCs as starting cells (Fig. 1). After differentiation, the resultant cells were subjected to immunofluorescence assays with cell specific antibodies: beta III-tubulin, Gal-C and GFAP primary antibodies for neurons, oligodendrocytes and astrocytes, respectively (Fig. 2). The results clearly indicated the presence of neurons, oligodendrocytes and astrocytes in the differentiated NSCs. However, no signal was obtained using microglia-specific antibodies, CD11b, indicating the inability of NSCs to form microglia (Fig. 2), consistent with previous reports [39].

### GAA Repeats are Stable in YG8R Fibroblasts, NSCs and Differentiated NSCs

**Figure 3:**
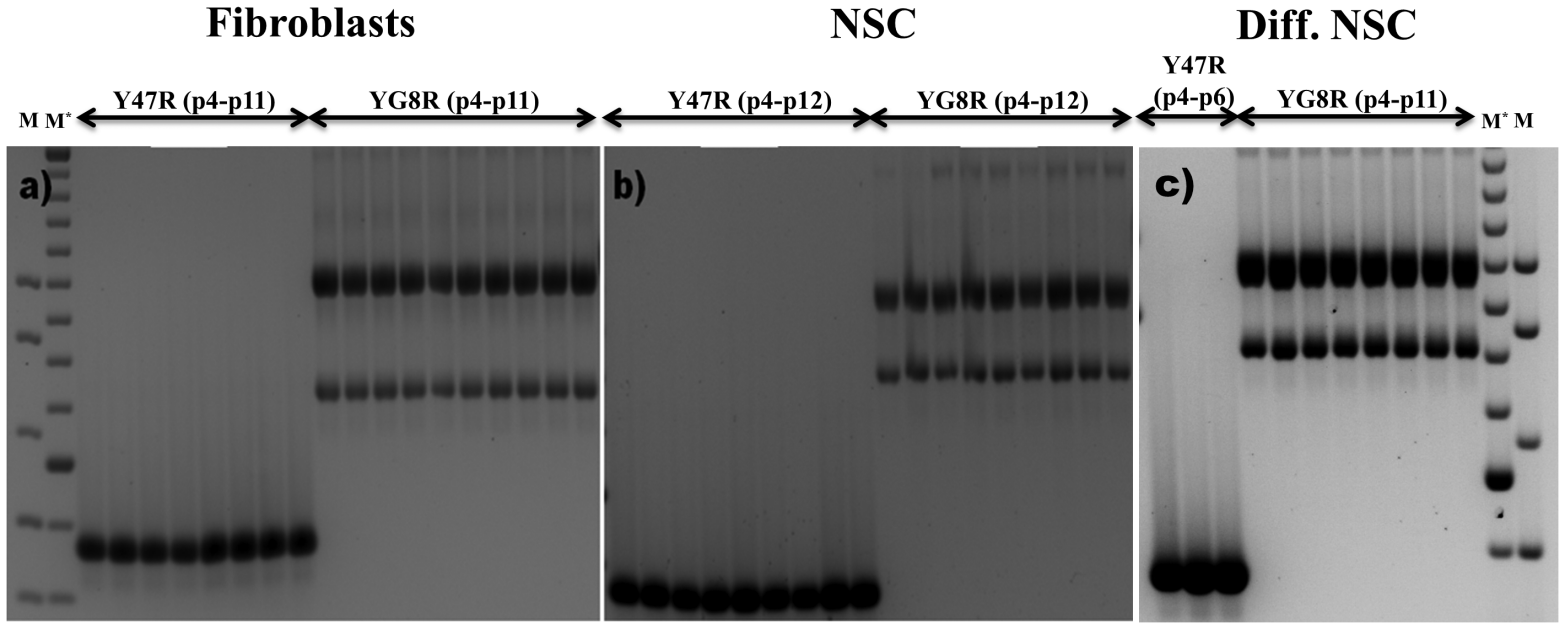
GAA repeat instability analysis. Ethidium bromide-stained agarose gels showing inverted images of GAA repeat PCR products obtained from the successive passages of Y47R and YG8R cells: **a**) primary fibroblasts (p4-p11), **b**) NSCs (p4-p12), and **c)** differentiated NSCs obtained from NSCs (p4-p11). M = 1 kb plus DNA marker, M* = 100 bp DNA marker.

**Figure 4:**
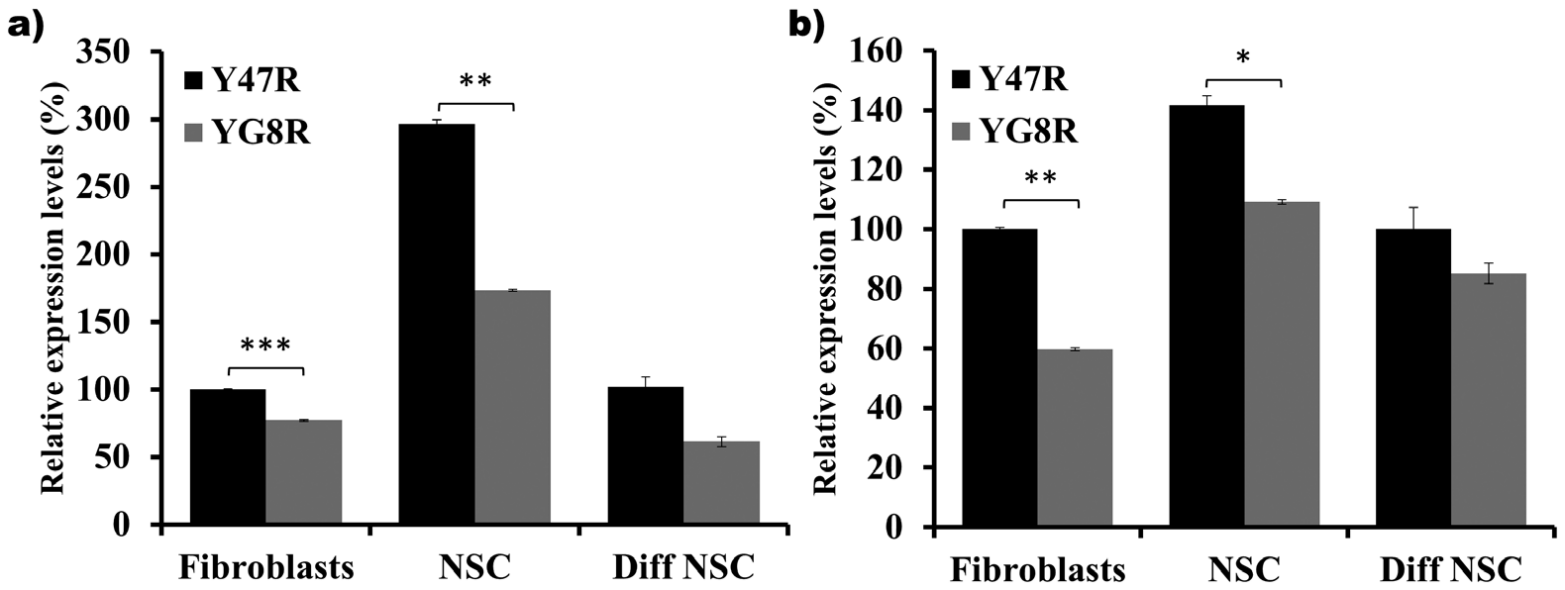
Frataxin expression levels. (**a**) *FXN* mRNA expression was analyzed by qRT-PCR of Y47R and YG8R fibroblasts, NSCs and differentiated NSCs. The mean values of NSCs and differentiated NSCs data are normalized to the mean *FXN* mRNA level of the Y47R fibroblasts taken as 100%. Two individual cDNA samples were analyzed for each cell type and each reaction was carried out in triplicate. Values were expressed relative to both *Gapdh* and *β2M* expression levels. **b**) Frataxin protein levels were assessed by frataxin dipstick assay of cell lysates and values were expressed relative to the signal intensity of the goat-anti-mouse antibody (GAM). Each reaction was carried out in triplicate. Error bars represent s.e.m (*p<0.05, **p<0.01, ***p<0.001).

Non-interrupted FRDA GAA repeats are known to be dynamic, exhibiting both intergenerational and somatic instability. Thus, non-pathogenic parental premutations can be transmitted to offspring as expanded pathogenic GAA repeats [40], while age-related GAA hyperexpansion has been detected particularly in DRG and cerebellum tissues [41]. We have previously shown that YG8R mice, which contain non-interrupted GAA repeats, also exhibit both somatic intergenerational and somatic instability, particularly in the DRG [14], [42]. Therefore, we were interested to determine if cells derived from such an animal showed GAA repeat instability when grown in culture. PCR analysis of the *FXN* GAA repeats from cells isolated from 4–11 consecutive passages of YG8R fibroblasts (Fig. 3a) or 4–12 consecutive passages of NSCs (Fig. 3b) showed no GAA repeat instability. Furthermore, differentiated NSCs obtained from 4–11 consecutive passages of NSCs also showed no GAA repeat instability (Fig. 3c). This is consistent with previous reports where GAA repeat instability has only been observed in iPS cells derived from skin fibroblasts of FRDA patients, but not in the fibroblasts themselves nor in the NSCs derived from the iPS cells [30], [32]. As expected, the control Y47R-derived fibroblasts, NSCs and differentiated NSCs also did not show any GAA repeat instability.

### Reduced Frataxin mRNA and Protein Levels in YG8R Mouse Cells

Since FRDA is characterised by reduced levels of frataxin mRNA and protein, we have quantified the frataxin mRNA and protein levels in the Y47R and YG8R mouse derived fibroblasts, NSCs and differentiated NSCs. *FXN* mRNA expression analysis of YG8R cells revealed a 23% reduction in fibroblasts (p<0.001), 42% reduction in NSCs (p<0.001) and a non-significant 41% decrease in the differentiated NSCs compared to Y47R cells (Fig. 4a). Similarly, quantification of frataxin protein YG8R levels also revealed reduced levels of frataxin protein in fibroblasts (40%, *p*<0.05), NSCs (23%, *p*<0.05) and differentiated NSCs (15%, ns) compared to Y47R mouse cells (Fig. 4b). This indicates that the expanded GAA repeats impair *FXN* transcription in all YG8R cultured cell types.

### Increased *FAST1* Levels in YG8R Mouse Primary Fibroblasts

**Figure 5:**
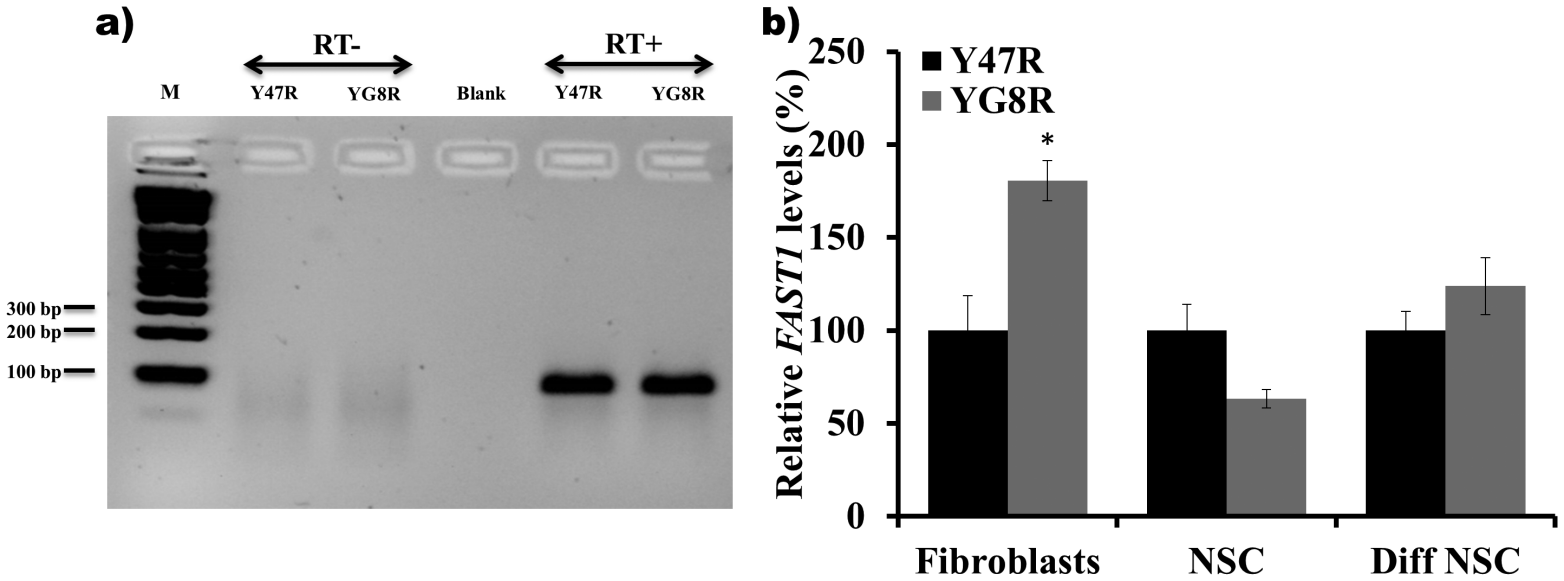
*FAST1* expression analysis in Y47R and YG8R cells. **(a)** Strand specific RT-PCR using a FAST-RT primer showed the presence of an antisense transcript. The *FAST1* PCR product did not amplify in the absence of reverse transcriptase (RT-). **(b)** qRT-PCR analysis of *FAST1* levels in fibroblasts, NSCs and differentiated NSCs showed increased levels of *FAST1* in fibroblasts but no statistical difference was detected in the other two cell types of YG8R cells compared to Y47R cells. Two individual cDNA samples were analyzed for each cell type and each reaction was carried out in triplicate. Values were expressed relative to both *Gapdh* and *Hprt* expression levels. Error bars represent s.e.m (*p<0.05). M = 1 kb plus DNA marker.

Antisense transcription has recently been proposed to have an important role in regulation of sense gene expression. Recent evidences have also been suggested that the antisense transcripts are associated with number of TNR expansion diseases such as HD [43], FRAXA [44], [45], SCA7 [46], SCA8 [47] and DM1 [48]. Recently, De Biase and colleagues [35] reported that frataxin antisense transcript 1 (*FAST1*) levels are significantly increased in human FRDA primary fibroblast cells and are associated with depletion of CCCTC-binding factor (CTCF), suggesting the involvement of these *cis-* and *trans*-acting elements in the transcriptional repression of the *FXN* gene [35]. To examine *FAST1* expression levels in Y47R and YG8R cells, we performed strand-specific cDNA synthesis of *FAST1* (Fig. 5a) followed by qRT-PCR analysis. In agreement with the findings by De Biase and colleagues [35], *FAST1* levels showed significant increase in YG8R primary fibroblasts (183%, *p*<0.05). However, YG8R NSCs and differentiated NSCs did not show a significant change in *FAST1* levels compared to Y47R cells (Fig. 5b), suggesting that effects on *FAST1* expression may be cell-type selective.

### Increased DNA Methylation in YG8R Mouse Cells

**Figure 6:**
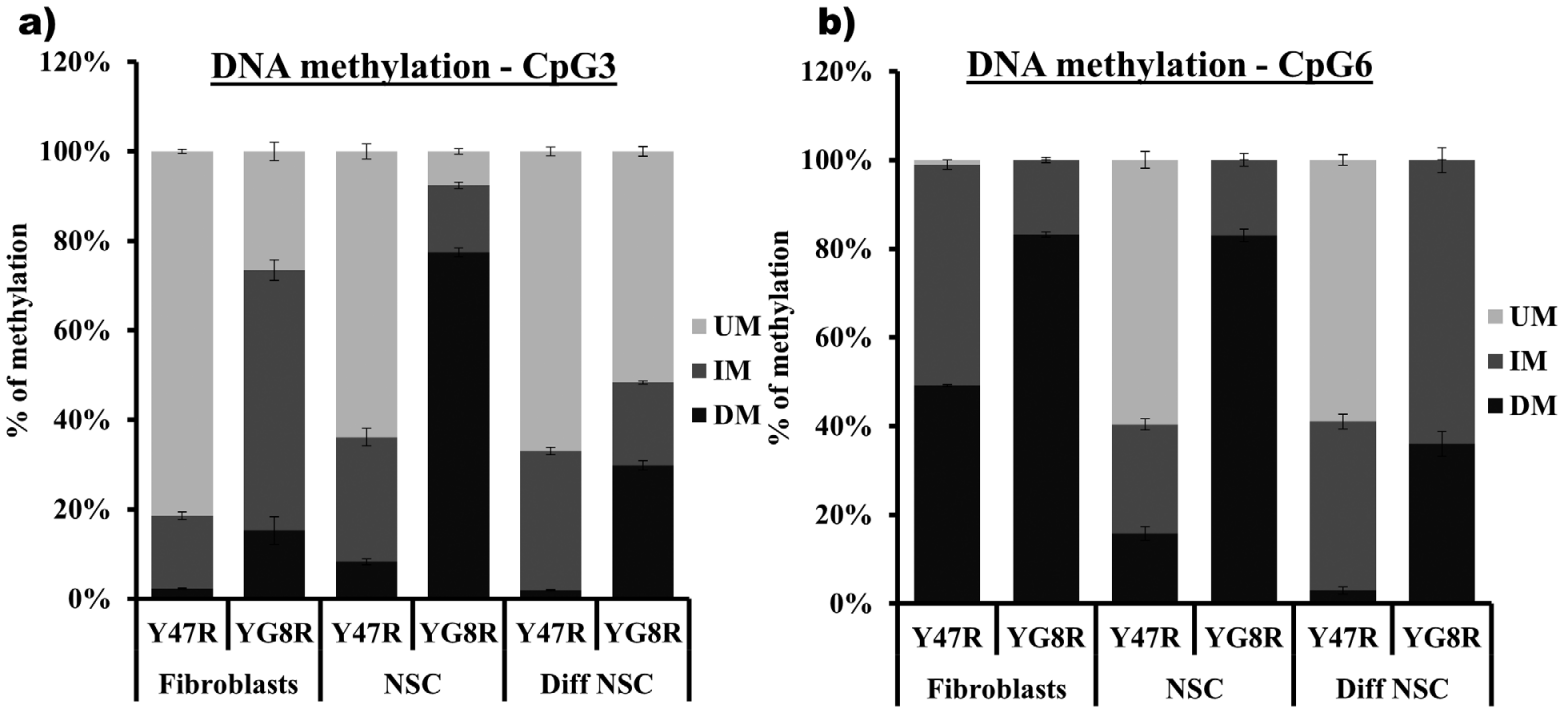
DNA methylation analysis. MethylScreen analysis of two CpG sites, CpG3 and CpG6, in the *FXN* upstream GAA repeat region of DNA from Y47R cells and YG8R cells. UM = unmethylated, IM = intermediately methylated, DM = densely methylated. The experiment was repeated twice for each cell type and each reaction was carried out in duplicate. Error bars = s.e.m.

The *FXN* transcriptional silencing mechanism in FRDA is not yet fully understood. However, recent evidence indicates that an epigenetic abnormality is involved [49]–[51]. Therefore, we have quantified the degree of DNA methylation in the Y47R and YG8R mouse cells by MethylScreen assay [37] at two CpG sites of the *FXN* upstream GAA repeat region, designated CpG3 and CpG6 [38]. CpG3 and CpG6 sites have previously been identified as two significantly differentially methylated sites in FRDA versus control lymphoblastoid cells [49]. Our results from fibroblasts, NSCs and differentiated NSCs reveal an increase in DNA methylation at both CpG sites in YG8R cells compared with control Y47R cells. In fibroblast cells we found that densely methylated (DM) values increased from 2.2% to 15.3% at CpG3 (p<0.01) and from 48% to 83% at CpG6 (p<0.001) (Fig. 6). Similarly, the DNA methylation levels were significantly increased in NSCs with DM values increasing from 8.3% to 77.4% at CpG3 (p<0.001) and from 15% to 83% at CpG6 (p<0.001) (Fig. 6). The results for differentiated NSCs have also shown a significant increase in DNA methylation at the two sites studied in YG8R cells compared to Y47R cells, with DM values increasing from 1.9% to 29.8% at CpG3 (p<0.001) and from 3% to 36% at CpG6 (p<0.001) (Fig. 6). These results are in agreement with recently published data using YG8R mouse heart and cerebellum tissue [50], suggesting that these cells will serve as a useful cell culture system in which to test the potential epigenetic-based frataxin-increasing compounds.

### YG8R Fibroblasts, NSCs and Differentiated NSCs Show Less Tolerance to Induced Oxidative Stress

**Figure 7:**
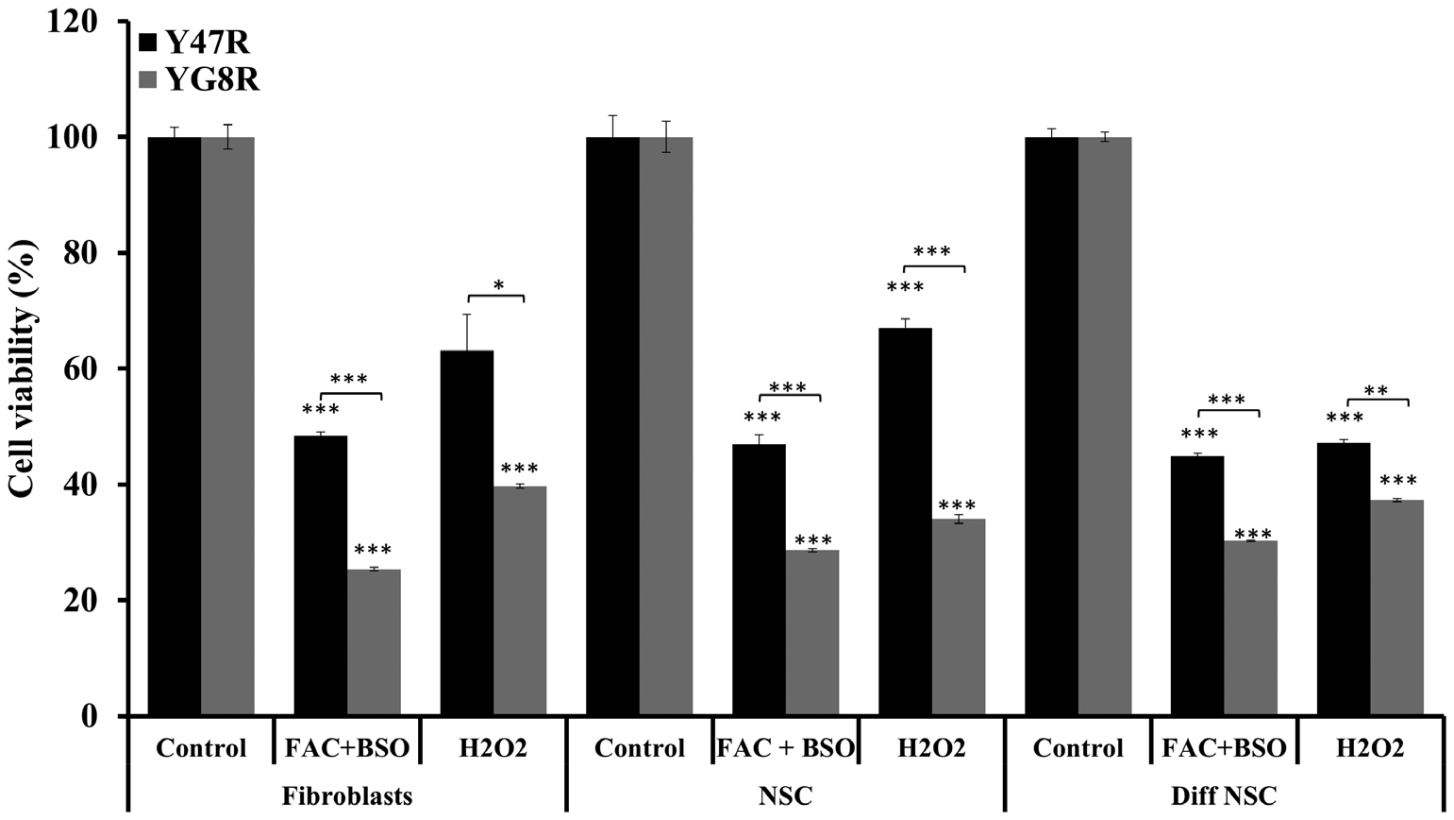
Susceptibility to oxidative stress in Y47R and YG8R cells. Treatment of Y47R and YG8R cells with 100 µM H_2_O_2_ or FAC (100 µg/ml) and BSO (1 mM) significantly reduced the cell viability in all cell types, but to greater extent in YG8R cells compared to Y47R cells. The experiment was repeated twice for each cell type and each reaction was carried out as 3–6 replicates. Error bars represent s.e.m (*p<0.05, **p<0.01, ***p<0.001).

Biochemical studies of mitochondrial enzymes have suggested that FRDA pathology may be, at least in part, caused by oxidative stress due to and mitochondrial dysfunction. Endomyocardial biopsies from two unrelated FRDA patients showed deficient activity of Fe-S containing enzymes, but normal enzyme activities in skeletal muscle and lymphocytes [52]. Also, human fibroblasts derived from FRDA patients showed sensitivity to hydrogen peroxide induced oxidative stress [21]. In addition, the YG8R FRDA YAC transgenic mouse model also exhibited signs of oxidative stress [34]. Therefore, we were curious to investigate the extent of oxidative stress in our YG8R derived fibroblasts, NSCs and differentiated NSCs. Treatment of YG8R mouse primary fibroblasts, NSCs and differentiated NSCs with H_2_O_2_ for 48 hours resulted in reduced cell viability, compared to Y47R mouse cells (Fig. 7). Although the Y47R mouse cells also showed a significant reduction in cell viability, the reduction in cell viability in YG8R mouse cells was more pronounced, indicating that these cells have less tolerance to oxidative stress. We then treated the cells with a combination of 100 µg/ml FAC and 1 mM BSO for 48 hours followed by assessing the cell viability by PrestoBlue® assay. Consistent with our H_2_O_2_ results, FAC and BSO treatment of YG8R mouse cells also showed a significant reduction in cell viability compared to Y47R mouse cells (Fig. 7). This reduction in cell viability was significant in all cell types.

### Reduced Aconitase Activity and *Pgc-1α* Expression in the YG8R Mouse Cells

**Figure 8:**
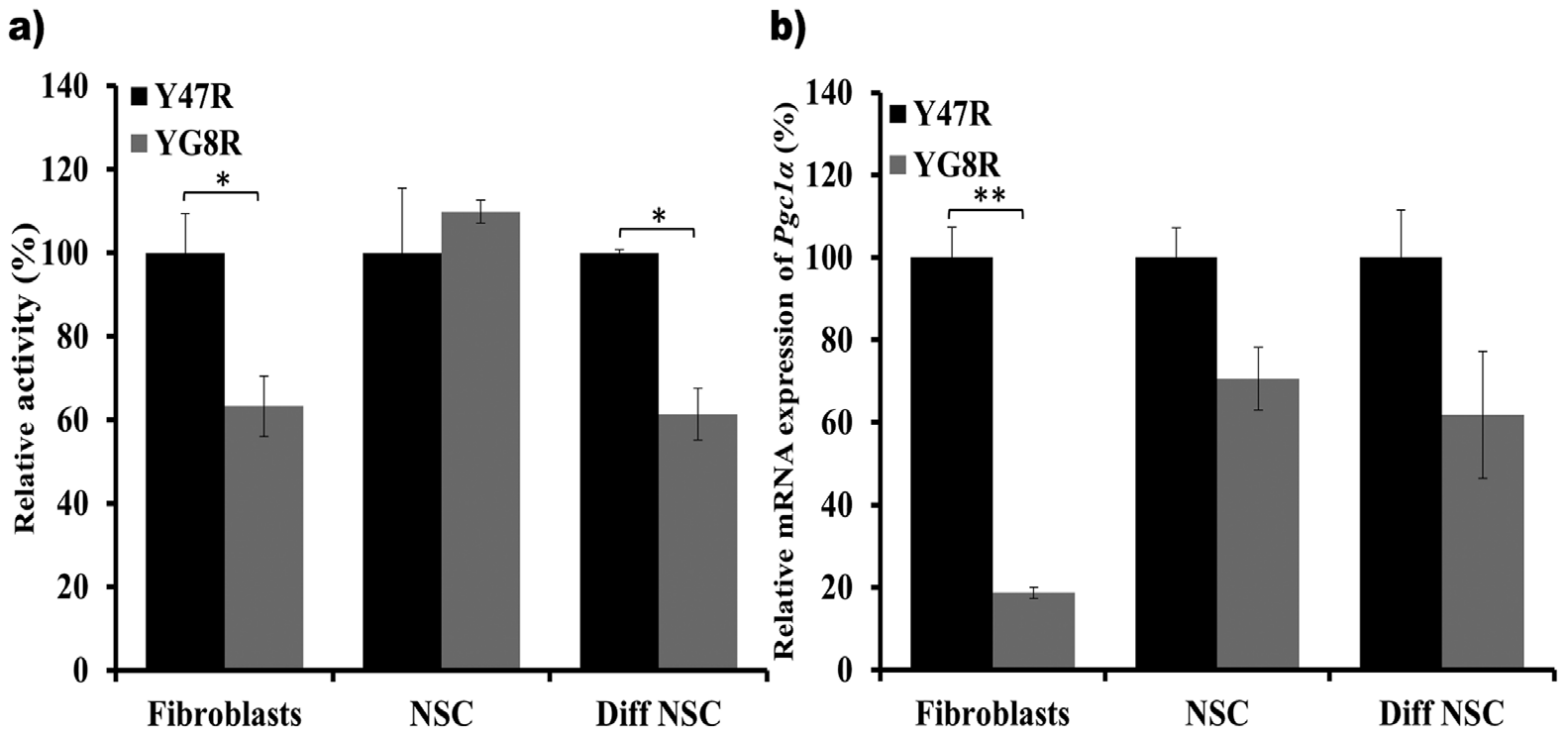
Aconitase activity and *Pgc-1α* expression levels in Y47R and YG8R cells. (**a)** Aconitase activities for Y47R and YG8R mouse cell samples. The experiment was performed twice on two samples in triplicate with values being calculated relative to citrate synthase activity. Values were expressed relative to the Y47R mouse cell value set at 100%. (**b)** *Pgc-1α* expression levels in Y47R and YG8R cells were quantified by qRT-PCR analysis using both *Gapdh* and *β2M* as endogenous controls. The mean values of YG8R data are normalized to the mean *Pgc-1α* mRNA level of the Y47R cells set at 100%. In each experiment, two individual cDNA samples were analyzed for each cell type and carried out in triplicate. Error bars represent s.e.m (*p<0.05, **p<0.01).

It has been reported that FRDA patient and mouse model tissues exhibit impaired activities of several Fe-S containing enzymes, such as aconitase and mitochondrial chain complexes (MRC) I, II, and III [17], [53]. Therefore, we have investigated the aconitase enzyme activity in the fibroblasts, NSCs and differentiated NSCs of Y47R and YG8R mice. Our findings showed significantly decreased aconitase activity in the YG8R mouse fibroblasts (37%, *p*<0.05) and differentiated NSCs (39%, *p*<0.05) compared to Y47R mouse cells (Fig. 8a). However, NSCs showed no difference in aconitase activity between Y47R and YG8R mouse cells. This could be due to the comparatively higher levels of frataxin expression observed in NSCs compared to fibroblasts and differentiated NSCs (Fig. 4).

Peroxisome proliferator activated receptor gamma (PPAR-γ) coactivator 1α (*Pgc-1α*) is a transcriptional coactivator that is a central inducer of mitochondrial biogenesis in cells. It has recently been reported that *Pgc-1α* expression is downregulated when the *FXN* expression is specifically inhibited by shRNA in human FRDA fibroblasts [54], [55]. In addition, increased *FXN* expression was achieved by PPAR-γ agonist treatment of FRDA cells [56]. These studies indicate close connection between *Pgc-1α* and *FXN* expression. Since our YG8R mouse derived cells show reduced levels of frataxin expression, we also quantified *Pgc-1α* mRNA expression in Y47R and YG8R mouse cells and found that *Pgc-1α* expression was significantly reduced by 82% (*p*<0.01) in the YG8R mouse fibroblasts compared to the Y47R cells (Fig. 8b). This result is in agreement with previous observations using human FRDA primary fibroblasts [55]. Although the *Pgc-1α* mRNA expression levels were reduced by 25% in the NSCs and 37% in differentiated NSCs, the levels of reduction did not reach statistical significance.

### Evaluation of Antioxidant Gene Expression Levels in YG8R Mouse Cells

**Figure 9:**
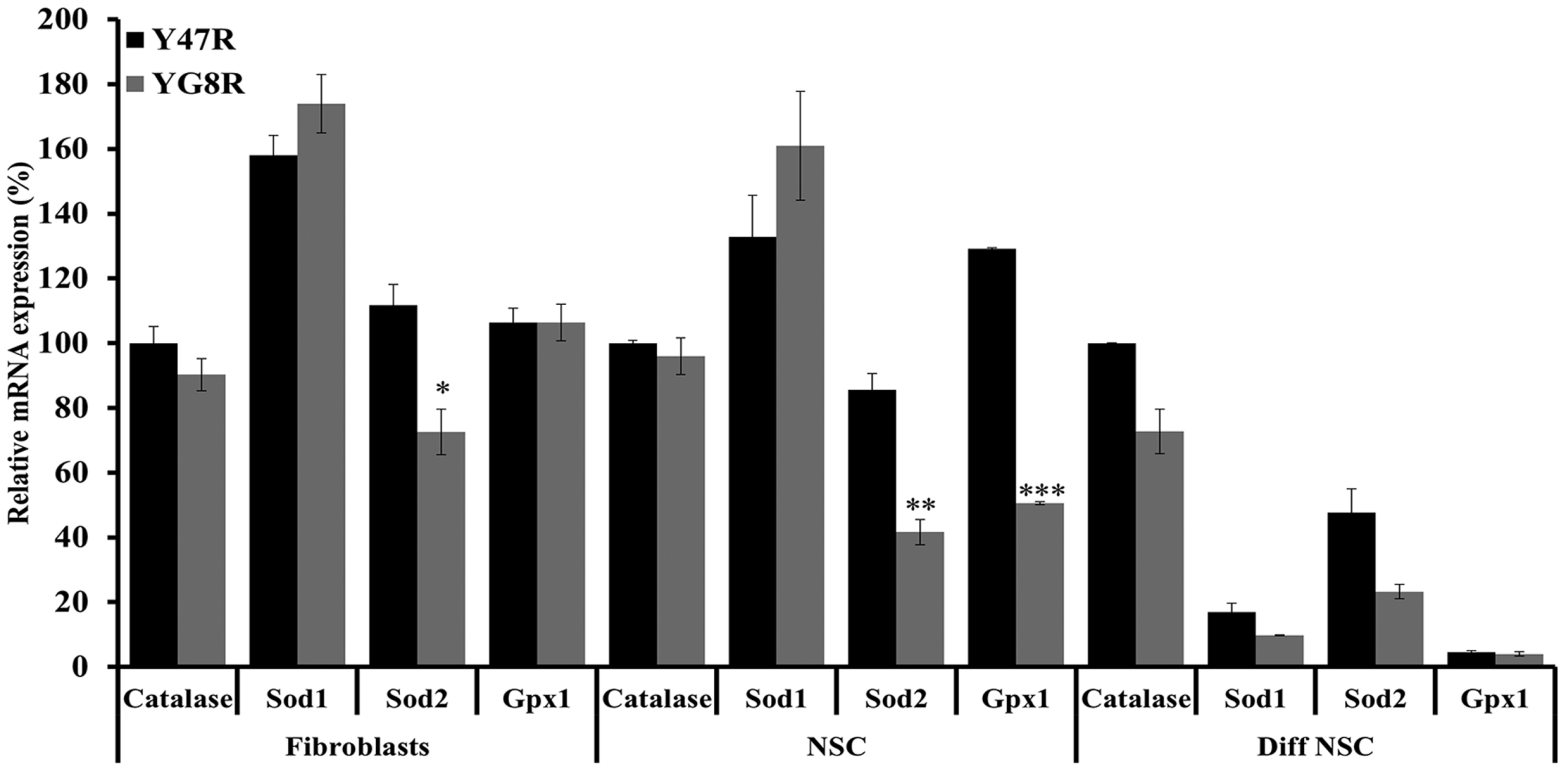
Antioxidant gene expression analysis. *Catalase*, *Sod1*, *Sod2* and *Gpx1* gene expression levels were determined in all three Y47R and YG8R cell types by qRT-PCR. Values were expressed relative to the levels of both *Gapdh* and *β2M* and in each case all gene expression levels were normalized to the mean expression levels of the *Catalase* gene of Y47R cells set at 100%. Two individual cDNA samples were analyzed for each cell type and each reaction was carried out in triplicate. Error bars represent s.e.m (*p<0.05, **p<0.01, ***0.001).

Reduced levels of *Sod1* (CuZn-SOD), *Sod2* (Mn-SOD) and *Gpx1* mRNA levels have previously been identified in YG8R mouse DRG, associated with decreased *Nrf2* expression [57], and also in *Pgc-1α* KO mice [58]. In addition, fibroblast cells derived from the *Pgc-1α* KO mice also show reduced levels of *Catalase* mRNA expression [59]. Similarly, downregulation of *FXN* and/or *Pgc-1α* in the FRDA patients’ fibroblasts and mouse models have shown significant decrease in the *Sod2* and other ROS antioxidant gene expression levels [34], [55], [60]. Furthermore, as we have shown here, YG8R mouse cells also exhibit reduced levels of *FXN* and *Pgc-1α* mRNA levels compared to Y47R control cells. Therefore, we decided to evaluate the antioxidant capacity in the YG8R cells compared to Y47R cells by quantifying mRNA levels of *Catalase*, *Sod1*, *Sod2* and *Gpx1*. We did not observe any significant differences in the *Catalase* and *Sod1* mRNA expression (Fig. 9). However, *Sod2* expression levels were significantly decreased in all three cell types studied in the YG8R mouse compared to Y47R mouse cells (Fig. 9). Furthermore, the *Gpx1* mRNA levels were downregulated, but only in the NSCs (*p*<0.001). These results further support the hypothesis that reduced expression of *FXN*, acting via decreased expression of *Pgc-1α* and/or *Nrf2*, may lead to reduced expression of several antioxidant genes, especially *Sod2*, which results in less tolerance to oxidative stress.

### Evaluation of DNA Mismatch Repair Gene Expression Levels in YG8R Mouse Cells

**Figure 10:**
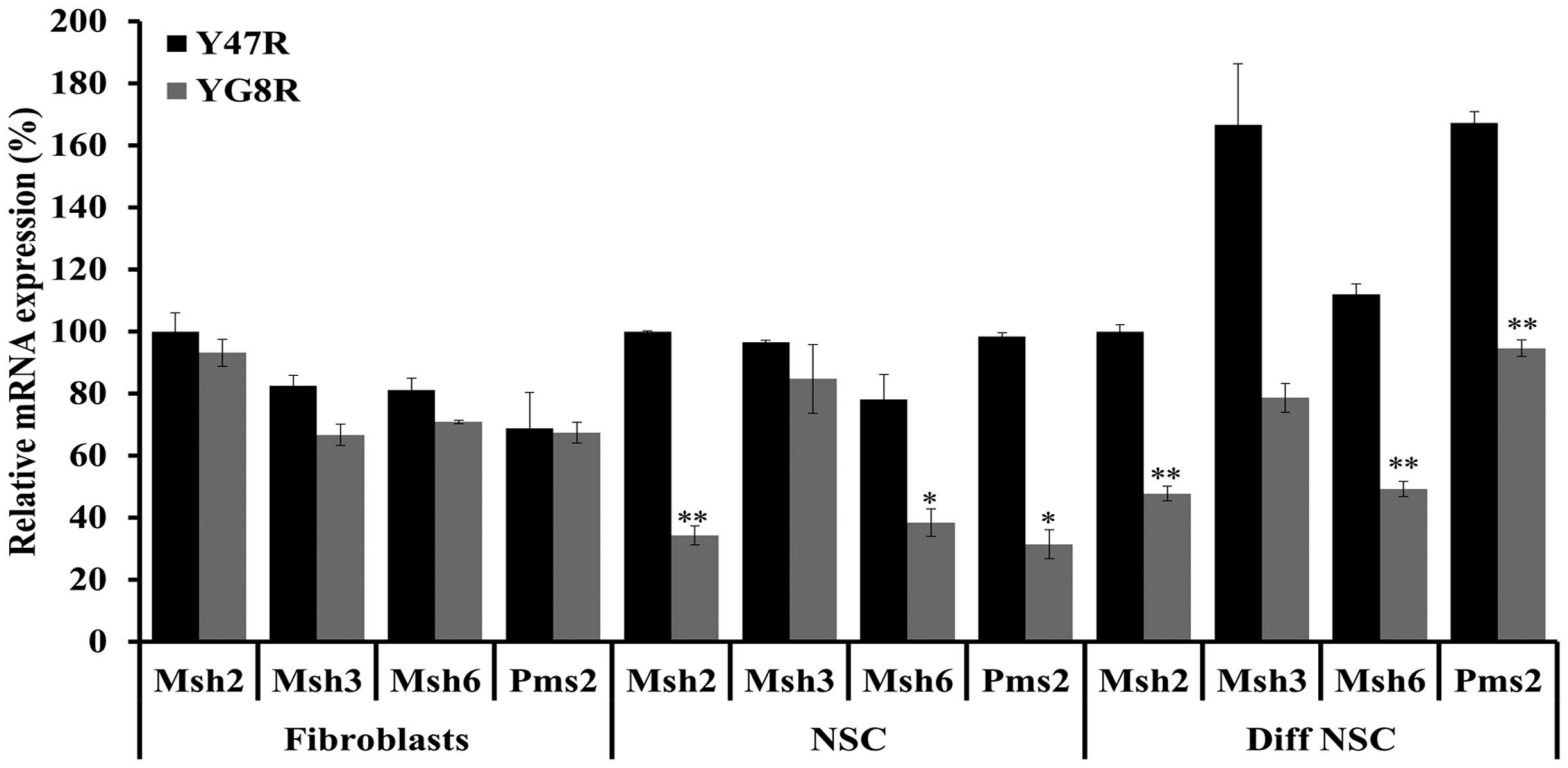
MMR gene expression levels. The expression levels of MMR genes, *Msh2*, *Msh3*, *Msh6* and *Pms2* were analyzed by qRT-PCR in all three cell types. Values were expressed relative to the levels of both *Gapdh* and *β2M* and in each case all gene expression levels were normalized to the mean expression levels of *Msh2* gene of Y47R cells set at 100%. Two individual cDNA samples were analyzed for each cell type and each reaction was carried out in triplicate. Error bars represent s.e.m (*p<0.05, **p<0.01).

It has recently been suggested that DNA mismatch repair (MMR) enzymes may play a role in FRDA disease progression by affecting GAA repeat instability [14], [61], [62]. Furthermore, increased levels of *MSH2* expression in FRDA human iPS cells contribute to the instability of GAA repeats [30]. We have previously shown that the YG8R mice have exhibited both somatic and intergenerational instability of the GAA repeats *in vivo* [14], [42], [63]. However, the primary cultures fibroblasts and NSCs isolated from this mouse model did not show any GAA repeat instability (Fig. 3). Therefore, to identify the possible mechanism underlying the lack of GAA repeat instability in such cells we quantified the mRNA expression levels of *Msh2*, *Msh3*, *Msh6* and *Pms2* genes by qRT-PCR. Fibroblasts did not show any significant difference of MMR gene expression levels between YG8R and Y47R control mouse cells. However, YG8R NSCs showed significant reduction in *Msh2* (66%, p<0.01), *Msh6* (50%, p<0.05) and *Pms2* (69%, p<0.05) expression levels compared to control Y47R NSCs (Fig. 10). Similarly, differentiated NSCs of YG8R mice also showed a significant downregulation of all four MMR genes compared to Y47R cells: such as *Msh2* (52%, p<0.01), *Msh3* (53%, p<0.05), *Msh6* (66%, p<0.01) and *Pms2* (44%, p<0.01). Therefore, the observed downregulation of MMR genes may contribute to the lack of GAA repeat instability in such cells.

## Discussion

We have generated and characterised novel FRDA mouse fibroblast and NSCs cell lines from YG8R mice, and we have shown that the NSCs are capable of differentiating into three neural cell lineages: neurons (10–15%), oligodendrocytes (15–20%) and astrocytes (70–80%). In contrast to YG8R mouse tissues that have shown both intergenerational and somatic instability *in vivo* [42], [63], YG8R fibroblasts, NSCs and differentiated NSCs did not exhibit any GAA repeat instability over extensive passage numbers tested. Similar GAA repeat stability has been detected in human FRDA fibroblasts and iPSC-derived NSCs [30], [32], [64]. Although the mechanism underlying this lack of instability in these cells is not clear, it has been proposed that *MMR* gene expression levels may play vital role in GAA repeat instability as increased expression of *MSH2*, *MSH3* and *MSH6* levels have been associated with increased GAA repeat instability in human iPS cells [30], [32]. Also, shRNA knockdown of either *MSH2* or *MSH3* in a human cellular model slowed the rate of GAA repeat expansion [61]. Furthermore, ectopic expression of *MSH2* and *MSH3* in human primary fibroblasts has triggered GAA repeat expansion at the *FXN* gene locus [61]. An important finding in our model cell culture system is that YG8R NSCs and differentiated NSCs containing stable expanded GAA repeats show downregulation of several MMR genes. This finding supports there being a role for the MMR system in GAA repeat instability in FRDA.

To further characterise the YG8R mouse cells, we quantified the frataxin mRNA and protein expression levels at the *FXN* locus compared to control Y47R cells. We detected reduced levels of frataxin expression, indicating that expanded GAA repeats induce transcriptional silencing of the *FXN* gene as seen in FRDA patients. Consistent with previous FRDA fibroblast data [35], *FAST1* antisense transcript levels were found to be significantly elevated in YG8R fibroblasts. However, NSCs and differentiated NSCs displayed no significant differences in *FAST1* levels, indicating that the regulation of *FAST1* expression may be cell selective. We have previously shown that the YG8R mice displayed increased levels of DNA methylation at the upstream region of the GAA repeats in brain, heart and cerebellum tissues [50]. To evaluate this epigenetic mark in our YG8R cells, we quantified the DNA methylation status at two CpG sites of the *FXN* upstream GAA repeat region, designated CpG3 and CpG6 [38]. Our analysis revealed a significant increase in DNA methylation in all three YG8R cell types at both sites, consistent with previous results using FRDA lymphoblastoid cells [49], FRDA patient tissues and primary cells [50], [65], [66] and FRDA mouse models [50]. Increased DNA methylation in these cells further strengthens the hypothesis that expanded GAA repeats exhibit heterochromatin mediated silencing of *FXN* gene.

It has been well documented that FRDA patient cells, mouse models and cell models with reduced frataxin levels exhibit increased susceptibility to oxidative stress [21,34,60,see review 67], although the exact mechanism for this effect is yet to be elucidated. In line with these findings, our YG8R mouse cells showed significantly reduced cell viability upon the exposure to H_2_O_2_ or FAC and BSO indicating that reduced frataxin levels may contribute to reduced efficiency of the oxidative stress defence mechanism. To further investigate the oxidative stress phenotype in our cells we have assessed the expression levels of a panel of antioxidant genes and found that *Sod2* mRNA expression was consistently reduced in all three cell types. This data suggest that part of the toxic mechanism involving oxidative stress in FRDA may be due to the deficient levels of *Sod2* expression as hemizygous *Sod2* (50% expression) mice have been shown to display increased oxidative damage to DNA and increased incidence of cancer [68]. Our findings are also consistent with previous studies that have shown significantly decreased *Sod2* expression levels in human primary fibroblasts [55], [60] and DRG tissue of YG8R mice [57]. *Pgc-1α* has been emerged as one of the master regulators of mitochondrial biogenesis. The role of this gene in FRDA pathogenesis is controversial as some results have shown downregulation of *Pgc-1α* [54], [55], whereas others have shown upregulation of *Pgc-1α* mRNA levels [60], in FRDA patient derived cells. However, FRDA patient fibroblasts with largest repeat sizes have shown complete loss of *Pgc-1α* expression in this latter study [60]. These results have led to the speculation that *Pgc-1α* expression may be dependent on several factors including the length of the GAA repeat size, cell type and age of onset. Our three YG8R mouse cell types show decreased levels of *Pgc-1α* expression, although the most significant reduction was found in fibroblasts, supporting the notion of cell-type variability. *Pgc-1α* activates nuclear-encoded genes required for mitochondrial biogenesis by co-activating several transcription factors, mainly *NRF-1*, *NRF-2* and *ERRα*. Consistent with these findings, it has been recently reported that YG8R mice have shown deficiency of *Nrf2* expression in the DRG tissue [57]. This downregulation of *Nrf2* is directly correlated with the *FXN* expression, indicating the involvement of multiple complex pathways in FRDA disease progression. Furthermore, our findings support the use of *Pgc-1α* or *Nrf2* activators as potential FRDA therapeutic strategies.

The deficiency of Fe-S cluster-containing enzyme activities is another distinct and early hallmark of FRDA [53]. Selective impairment of MRC complexes I/II/III and aconitase have been demonstrated in heart biopsies from FRDA patients with hypertrophic cardiomyopathy [17], [52], in lymphocytes [69] and in mouse models [34], [53]. It has also been suggested that frataxin acts as an iron chaperon in converting oxidative damaged (3Fe-4S) clusters into active (4Fe-4S) clusters of aconitase [70]. Our YG8R mouse fibroblasts and differentiated NSCs also showed reduced levels of aconitase activity, but no difference was detected in the NSCs, possibly due to the presence of comparatively higher levels of frataxin expression.

In conclusion, we have developed and characterised novel model cell culture systems that are derived from FRDA mice that contain either expanded GAA repeats (YG8R) or normal GAA repeats (Y47R). We show that the NSCs can differentiate into neurons, oligodendrocytes and astrocytes. Although the cells derived from the YG8R mice did not show any GAA repeat instability, they have displayed FRDA-like molecular phenotypes, including reduced frataxin expression levels, increased *FAST1* levels, increased susceptibility to oxidative stress and reduced aconitase activity. Furthermore, these cells also exhibit impairments in antioxidant *Pgc-1α* and MMR gene expression profiles. These novel NSCs and differentiated NSC cell models can be a valuable resource for investigating FRDA molecular disease mechanisms and for preclinical testing of novel FRDA therapies.
